# Endoscopic pancreatic duct stenting for pain palliation in selected pancreatic cancer patients: a systematic review and meta-analysis

**DOI:** 10.1093/gastro/goab001

**Published:** 2021-02-03

**Authors:** Pradeep K Siddappa, Fadi Hawa, Larry J Prokop, M Hassan Murad, Barham K Abu Dayyeh, Vinay Chandrasekhara, Mark D Topazian, Fateh Bazerbachi

**Affiliations:** 1 Division of Gastroenterology and Hepatology, Stanford University School of Medicine, Stanford, CA, USA; 2 Department of Internal Medicine, St. Joseph Mercy Ann Arbor Hospital, Ypsilanti, MI, USA; 3 Library Public Services, Mayo Clinic, Rochester, MN, USA; 4 Robert D and Patricia E Kern Center for the Science of Health Care Delivery, Mayo Clinic, Rochester, MN, USA; 5 Division of Gastroenterology and Hepatology, Mayo Clinic, Rochester, MN, USA; 6 Division of Gastroenterology, Massachusetts General Hospital and Harvard Medical School, Boston, MA, USA

**Keywords:** cancer-associated pain, Endoscopic retrograde cholangiopancreatography, palliative therapy, pancreas cancer, systematic review, meta-analysis

## Abstract

**Background:**

Abdominal pain is a debilitating symptom affecting ∼80% of pancreatic cancer (PC) patients. Pancreatic duct (PD) decompression has been reported to alleviate this pain, although this practice has not been widely adopted. We aimed to evaluate the role, efficacy, and safety of endoscopic PD decompression for palliation of PC post-prandial obstructive-type pain.

**Methods:**

A systematic review until 7 October 2020 was performed. Two independent reviewers selected studies, extracted data, and assessed the methodological quality.

**Results:**

We identified 12 publications with a total of 192 patients with PC presenting with abdominal pain, in whom PD decompression was attempted, and was successful in 167 patients (mean age 62.5 years, 58.7% males). The use of plastic stents was reported in 159 patients (95.2%). All included studies reported partial or complete improvement in pain levels after PD stenting, with an improvement rate of 93% (95% confidence interval, 79%–100%). The mean duration of pain improvement was 94 ± 16 days. Endoscopic retrograde cholangiopancreatography (ERCP)-related adverse events (AEs) were post-sphincterotomy bleeding (1.8%), post-ERCP pancreatitis (0.6%), and hemosuccus pancreaticus (0.6%). AEs were not reported in two patients who underwent endoscopic ultrasound-guided PD decompression. In the 167 patients with technical success, the stent-migration and stent-occlusion rates were 3.6% and 3.0%, respectively. No AE-related mortality was reported. The methodological quality assessment showed the majority of the studies having low or unclear quality.

**Conclusion:**

In this exploratory analysis, endoscopic PD drainage may be an effective and safe option in selected patients for the management of obstructive-type PC pain. However, a randomized–controlled trial is needed to delineate the role of this invasive practice.

## Introduction

Pancreatic cancer (PC) is the fourth leading cause of cancer-related death in the USA [1]. Fewer than 20% of patients are considered surgical candidates, while the majority are often treated with palliative interventions [2, 3].

Although PC is commonly asymptomatic in the early stages despite upstream biliary and/or pancreatic ductal dilatation [4–6], abdominal pain is considered among the most debilitating symptoms, afflicting ∼80% of PC patients, with approximately half of them requiring substantial opioid analgesia [7]. Pain affects the patient’s quality of life and influences performance status [8]. However, concerns have been raised regarding the frequent use of opioids in this context due to potentially shortened survival [9]. This decrease in survival has also been shown when other techniques were implemented for pain control, such as celiac plexus neurolysis (CPN) [10]. Moreover, CPN may not be more effective than modern opioid therapy in managing these symptoms [11].

The two principal mechanisms of PC abdominal pain are ductal hypertension secondary to obstruction and neuropathy secondary to neoplastic infiltration of regional nerves [3]. These mechanisms may result in distinctive pain patterns: neuropathic pain may be constant, while ductal obstruction may result in pain that worsens after meals.

Given the importance of palliation in PC patients and safety concerns for traditional pain control techniques, endoscopic pancreatic duct (PD) hypertension alleviation, via stenting, was hypothesized to benefit a subgroup of patients with obstructive-type post-prandial pain [12, 13]. Although relief of ductal obstruction is an effective treatment for select patients with painful chronic pancreatitis, the role of this technique remains unclear in cancer pain and thus has not been widely adopted [14, 15]. In this systematic synthesis of evidence, we aimed to evaluate the efficacy and safety of endoscopic PD decompression in patients with obstructive-type PC pain and whether current pain palliation options should be broadened to include PD stenting in a defined subgroup of patients.

## Materials and methods

This systematic review was reported following the ‘preferred reporting items for systematic reviews and meta-analyses’ (PRISMA) with an *a priori* protocol [16]. 

### Data sources and search strategies

A medical reference librarian conducted an extensive search of multiple databases without any language restriction from the inception of the database to 7 October 2020. The data sources and search strategies are provided in **Supplementary File 1**. 

### Inclusion criteria

The inclusion criteria consisted of randomized–controlled trials (RCTs), experimental studies, and non-randomized observational clinical studies, including case reports and case series that reported the use of endoscopic retrograde cholangiopancreatography (ERCP) or endoscopic ultrasound (EUS) in PD decompression to alleviate PC-associated abdominal pain. This type of pain is defined as upper abdominal pain in the setting of main PD obstruction with or without documentation of post-prandial exacerbation of pain. In the case of multiple publications emanating from the same center at different periods, we elected to include the most recent report to avoid duplication of patients.

While obstructive-type abdominal pain is mainly post-prandial in nature, this characteristic was rarely specified. Accordingly, a general definition of PC-associated pain was adopted to capture all relevant studies, which underwent a rigorous appraisal and methodological quality assessment.

### Exclusion criteria

We excluded duplicated studies, reviews, animal/*in vitro* studies, non-endoscopic PD decompression, and those with insufficient clinical data. Patients who met the American College of Gastroenterology criteria for acute pancreatitis were excluded [17].

### Data extraction

Two independent reviewers (P.S. and F.H.) selected the studies and extracted the relevant data onto a standardized form. Data included the year of publication, country of origin, publication language, publication format (full-text article, letter to the editors, abstract form), type of study (RCT, experimental study, case report, case series, prospective observational study), number of patients, mean age, sex, tumor location, baseline symptoms, radiological findings before the intervention (ductal dilation, strictures), pain characteristics, clinical reasoning (gestalt) to patient selection, type of intervention (ERCP or EUS-based), technical success, type of stent if placed (plastic, metal), length of the stent, adverse events (AEs), symptom improvement (subjective improvement of pain, improvement of pain via standardized scales), mean rate and duration of pain improvement post intervention, and follow-up period.

### Main outcomes

Given that pain is always a subjective phenomenon [18], regardless of quantifying scales, the primary outcome was any pain improvement post intervention. Secondary outcomes included ‘time to improvement’ of pain after the procedure, the mean duration of pain improvement after each pancreatic intervention, and pain improvement via standardized scales.

### Assessment of methodological quality of included studies

The same two reviewers assessed the methodological quality of included studies with discussion and adjudication by the corresponding author in case of disagreement. We used the methodological quality and synthesis of case series and case reports tool reported previously [19–25]. Accordingly, each study is evaluated based on four domains: the selection of study groups, ascertainment, causality, and reporting (Supplementary Table 1).

### Statistical analysis

When the included series provided information sufficient to produce a rate of pain improvement (i.e. a numerator of patients with improved pain and an explicit denominator), we estimated an event rate from each study. Studies contributing fewer than four patients were not included in the meta-analysis because an event rate cannot be calculated. The Freeman–Tukey double arcsine transformation was used to stabilize the variance and produce 95% confidence intervals (CIs) with sensitivity analyses using the logit transformation [26]. We pooled event rates across studies using the random-effects model [27]. For the continuous outcome of the duration of pain improvement, we extracted the mean duration from series in which patients had technical success along with the standard deviation. Missing standard deviations were imputed from the mean of reported deviations. We pooled the mean duration across studies using the random-effects model. Heterogeneity was assessed using the *I*-squared statistic and Cochrane *Q* test. Given the non-comparative design of the majority of the included studies and the heterogeneity between studies, publication bias was not evaluated using funnel-plot-based statistical tests [28]. *P *<* *0.05 was considered significant. Statistical analysis was done using Stata 16 software (StataCorp. 2019. Stata Statistical Software: Release 16. College Station, TX, USA: StataCorp LLC). The remaining outcomes were reported narratively.

## Results

### Study characteristics

**Figure 1.**
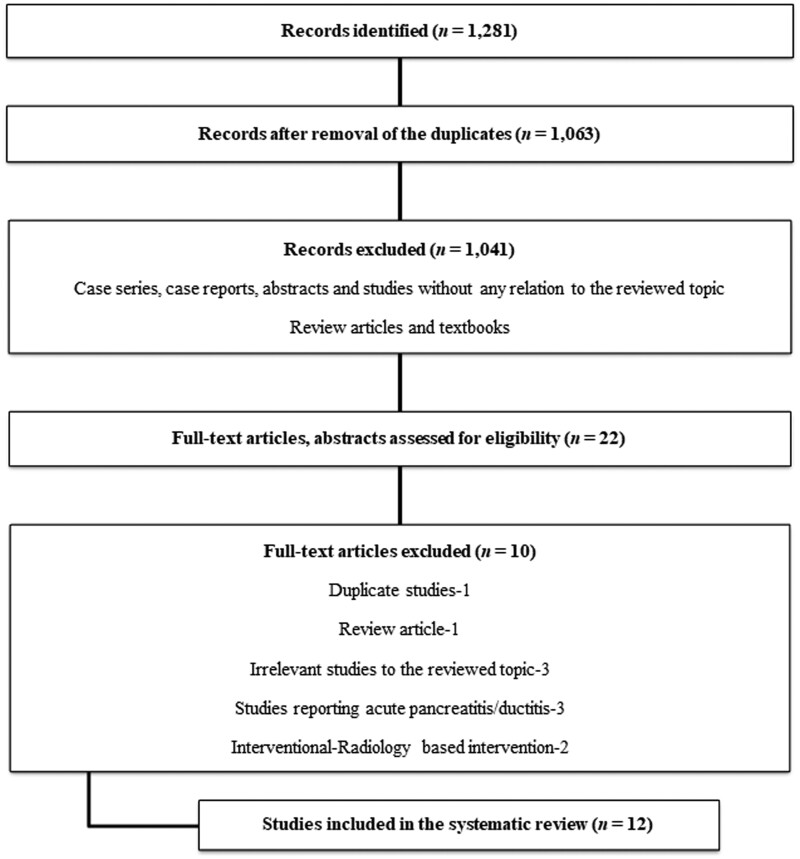
Flow diagram showing the different phases of the systematic review

Figure 1 shows the systematic-review flow. We identified 12 publications from five countries between 1989 and 2019 [3, 29–39]. One study was in Chinese and subsequently translated into English by a native speaker [29]. Three publications were in abstract form [30–32] and the remaining were full-text articles [3, 29, 33–39]. There were 3 case reports with a total of 4 patients [31, 33, 36], three retrospective case series reporting a total of 54 patients [3, 32, 35], and the remainder were prospective studies adding 134 patients [29, 30, 34, 37–39], thus totaling 192 patients with PC-associated abdominal pain. No RCTs were identified.

### Patient characteristics

We identified a total of 192 eligible patients (190 underwent ERCP and 2 underwent EUS), in whom PD stenting was successful in 167 (87.0%). Patients with PD stenting had a mean age of 62.5 years and 98 patients were males (58.7%) (Table 1).

**Table 1.** Main demographic characteristics of the studies included in the systematic review

First author/year	Country	Study design	Number of patients	Patients with technical success, *n* (%)	Mean age (years)^a^	Male, *n* (%)^a^
Harrison/1989 [33]	USA	Case report	1	1 (100%)	66	1 (100%)
Ashby/1995 [34]	USA	Prospective case series	5	5 (100%)	42	2 (40%)
Catalano/1998 [30]	USA	Prospective case series	19	15 (79.0%)	68	6 (40%)
Costamagna/1999 [3]	Italy	Retrospective case series	50	34 (68.0%)^b^	71.5	23 (67.6%)
Tham/2000 [38]	USA	Prospective case series	6	6 (100%)	64.5	1 (17%)
Wehrmann/2005 [39]	Germany	Prospective case series	20	19 (95.0%)	67.6	13 (68.4%)
Akbar/2012 [35]	USA	Retrospective case series	2^c^	2 (100%)	53.5	2 (100%)
Gao/2014 [37]	China	Prospective comparative study	42	38 (90.5%)	68.2	22 (57.9%)
Nair/2015 [31]	USA	Case report	2	2 (100%)	58.5	2 (100%)
Li/2016 [29]	China	Prospective comparative study	42	42 (100%)	58.6	26 (62%)
Ryabov/2017 [32]	NR	Retrospective case series	2^c^	2 (100%)	NR	NR
Abramyan/2019 [36]	USA	Case report	1	1 (100%)	69	0 (0%)

NR, not reported.

a Mean age and sex data are based on patients with abdominal pain who had technical success with placement of pancreatic duct (PD) stent.

b The reported technical success in Costamagna *et al.* [3] was 81.8% based on the total number of patients of 55. However, to fulfill our systematic-review-inclusion criteria, we only considered those patients with obstructive pain (*n *=* *50), for whom the sole intervention was PD stenting in 34 patients (patients who underwent concomitant intraluminal brachytherapy were excluded). Hence, the technical success rate is reported as 34/50 (68.0%).

c Case reports were deemed to be those with up to four patients and case series were deemed those with more than four patients (Abu-Zidan FM, Abbas AK, Hefny AF. Clinical ‘case series’: a concept analysis. *Afr Health Sci*. 2012 Dec; 12[4]:557–62). Only two patients in Akbar *et al.* [35] and Ryabov *et al.* [32] case series fulfilled the inclusion criteria.

### Clinical presentation

Clinical decision-making in patient selection for PD drainage is relayed in Supplementary Table 2. Post-prandial epigastric abdominal pain was described in 76 patients [3, 38, 39]. Epigastric and upper abdominal pain was mentioned in 46 patients [31, 33, 36, 37]. The remaining 70 patients had abdominal pain not otherwise specified [29, 30, 32, 34, 35]. Of the 12 studies, 11 (including 172 patients) assessed the PD diameter upstream to the malignant stricture, reporting ductal dilation in all patients (Catalano *et al.* [30] did not report this finding). When the location of malignancy was reported (56 patients), the most common location was the pancreatic head (45 patients) [3, 30, 33, 34, 36, 38, 39], followed by the body of the pancreas (9 patients) [30, 35] and the tail (2 patients) [30]. Tumor location was not specified in the remaining 136 patients. Sixty patients were on analgesic therapy prior to successful PD stent placement [3, 33, 38, 39].

### Procedures and AEs

Among 192 patients, 190 underwent an ERCP-guided PD stent placement attempt and 2 underwent EUS-guided decompression with PD stent placement (Table 2). Technical success was achieved in 167 out of 192 patients (87.0%; 165 patients via ERCP, 2 patients via EUS). The reasons for technical failure were failed cannulation, unsuccessful opacification of the PD, inability to negotiate the guide wire through the stricture, and inability to dilate the stricture. Criteria for pancreatic sphincterotomy performance were stated in one study, in which it was performed if the insertion of a 10 French (Fr) stent was attempted or if a 7 Fr stent was introduced without a prior biliary sphincterotomy [39]. Pancreatic sphincterotomy was explicitly reported in 40 patients [33, 35, 37] and stricture dilation in 154 patients [3, 29, 30, 37–39]. Pancreatic plastic stents were the most commonly used stents and were reported in 159/167 patients with a median size of 7 Fr (range, 5–11.5 Fr) and length of 7 cm. Six patients (6/167) underwent placement of pancreatic metal stents with a median diameter of 8 mm (range, 8–10 mm) and length of 60 mm [35, 36, 38]. The type of metal stents used were Viabil stents (Gore, Utica, NY, USA) in two patients [35], partially covered WallStents (Boston Scientific, Natick, MA) [38] in two patients, an uncovered Endocoil (Medtronic Instent, Minneapolis, MN, USA) in one patient [38], and a covered Wallflex (Boston Scientific, Natick, MA) in one patient [36]. In 2/167 patients, the type of pancreatic stent was not specified [31]. Concurrent biliary-stent placement was reported in 101/167 patients [29, 36–39]. A combination of EUS-guided CPN and EUS-guided PD decompression with plastic stent placement was performed in 2/167 patients [32].

**Table 2.** Main characteristics of procedures and subsequent pain response in the studies included in the systematic review

First author/year	Type of intervention	Technical success (%)	Upstream PD dilation^a^	Type of stent	Pain improvement rate post intervention, *n* (%)	Mean duration of response (days)	Mean follow-up duration (days)
Harrison/1989 [33]	ERCP	100%	Yes	Plastic	1 (100%)	30	150
Ashby/1995 [34]	ERCP	100%	Yes	Plastic	5 (100%)	69	132
Catalano/1998 [30]	ERCP	79%	NR	Plastic	12 (80.0%)	NR	300
Costamagna/1999 [3]	ERCP	68%	Yes	Plastic	30 (88.2%)	NR	NR
Tham/2000 [38]	ERCP	100%	Yes	Plastic (50%) SEMS (50%)	6 (100%)	170	170
Wehrmann/2005 [39]	ERCP	95%	Yes	Plastic	18 (94.7%)	105	105
Akbar/2012 [35]	ERCP	100%	Yes	SEMS	2 (100%)	94.5	510
Gao/2014 [37]	ERCP	90.5%	Yes	Plastic	26 (68.4%)	90	246
Nair/2015 [31]	ERCP	100%	Yes	NR	2 (100%)	NR	NR
Li/2016 [29]	ERCP	100%	Yes	Plastic	42 (100%)	90	180
Ryabov/2017 [32]	EUS	100%	Yes	Plastic	2 (100%)	NR	NR
Abramyan/2019 [36]	ERCP	100%	Yes	SEMS	1 (100%)	270	270

ERCP, endoscopic retrograde cholangiopancreatography; EUS, endoscopic ultrasound; NR, not reported; PD, pancreatic duct; SEMS, self-expanding metal stent.

a Evidence of pancreatic duct dilation on imaging prior to endoscopic intervention.

Repeat endoscopic intervention was explicitly reported in 16/167 patients due to stent occlusion (5 patients), migration (5 patients), and concurrent biliary-stent obstruction with cholangitis in the remaining 6 patients [29, 33, 39]. Harrison *et al.* [33] and Wehrmann *et al.* [39] reported exacerbated abdominal pain in two patients resulting from PD stent occlusion and migration, respectively, which were subsequently resolved after stent exchange. ERCP-related AEs (165 patients with ERCP) included post-ERCP pancreatitis (PEP) in 1/165 patients (0.6%) [37], post-sphincterotomy bleeding in 3/165 patients (1.8%, [2 biliary, 1 pancreatic]) [3, 39], and hemosuccus pancreaticus in 1/165 patients (0.6%) [3]. Patients who underwent EUS-guided decompression (two patients) were not reported to have any AEs. When PD stenting was successful (167 patients), stent-related AEs included stent migration in 6/167 patients (3.6%) [3, 29, 39] and stent occlusion in 5/167 patients (3.0%) [29, 33]. The incidence of procedure-related AEs was 8.8% (11/125 patients) in prospective studies and 11.9% (5/42 patients) in retrospective studies. No AE-related mortality was reported.

### Pain response and follow-up duration

**Figure 2.**
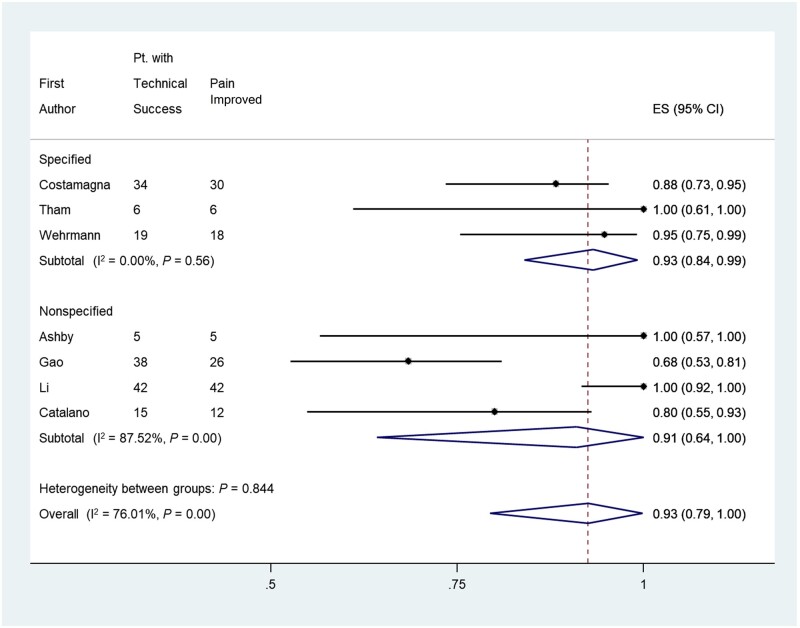
Forest plot of the effect sizes for the pain improvement rate in pancreatic cancer patients undergoing pancreatic duct stenting for obstructive-type pain; ES, effect size; Pt, patient; Specified, studies that specified post-prandial pain; Non-Specified, studies with non-specified abdominal pain. The diamond depicts the average of the effect sizes.

**Figure 3.**
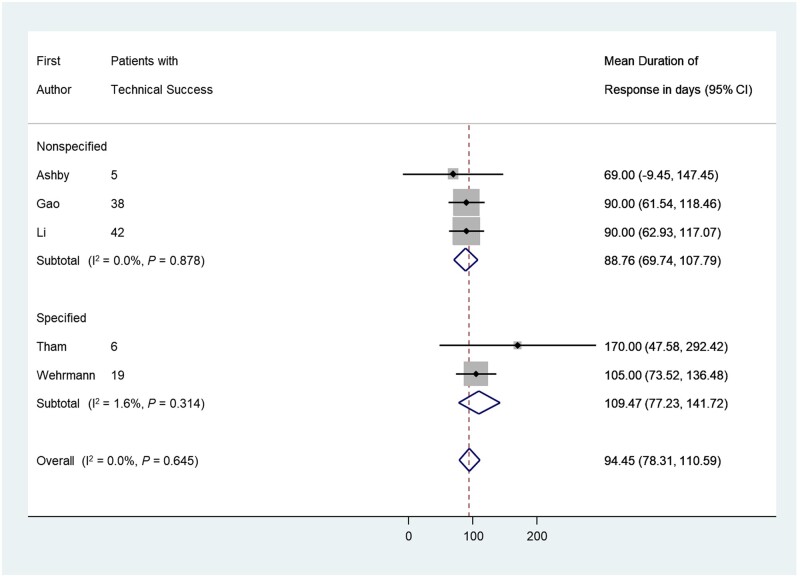
Forest plot of the effect sizes for the mean duration of response to pancreatic duct stenting in pancreatic cancer patients with obstructive-type pain; Specified, studies that specified post-prandial pain; Non-Specified, studies with non-specified abdominal pain. The diamond depicts the average of the effect size.

All included studies reported pain improvement, whether partial or complete, upon technical success (Table 2). Five studies (106 patients) prospectively applied symptom-scoring scales to measure the improvement in pain levels post intervention (4 studies employed a 10-point scale [29, 32, 37, 39] and 1 study employed a 4-point scale [34]). Of those, three studies utilized the visual analog scale with a statistically significant reduction in pain scores post intervention (*P *<* *0.05) (Supplementary Table 3) [29, 37, 39]. In the meta-analysis, the pain improvement rate based on subjective assessment (complete or partial) was derived from 159 patients and was 93% (95% CI, 79%–100%) (Figure 2) [3, 29, 30, 34, 37–39]. The pain improvement rate in the 59 patients with post-prandial epigastric abdominal pain was noted to be 93% (95% CI, 84%–99%) [3, 38, 39], whereas the remaining 100 patients had a pain improvement rate of 91% (95% CI, 64%–100%) [29, 30, 34, 37]. The time to improvement of symptoms was reported in three studies (seven patients) with a mean of 44 + 7 h [33, 34, 36]. Patients who were on analgesic therapy before the intervention were reported to have had a significant reduction in the analgesic dose or been taken off analgesic therapy completely [3, 33, 38, 39]. The overall mean duration of symptoms improvement after the initial intervention was ∼94 ± 16 days based on data from five studies (110 patients) (Figure 3) [29, 34, 37–39]. Patients were followed for a mean duration of 229 ± 117 days, based on data from nine studies (129 patients) [29, 30, 33–39].

Two studies reported a need for further interventions after PD stenting in some patients, for either symptom recurrence or cancer resection. These interventions included palliative double-bypass surgery (three patients) and classical Whipple procedure (one patient) [34], in addition to pylorus-preserving Whipple (one patient) [35].

### Assessment of the methodological quality of the included studies

**Figure 4.**
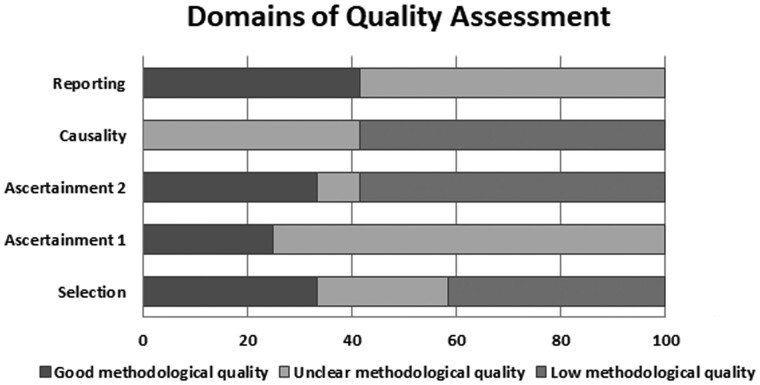
Evaluation of the methodological quality of the studies in the systematic review

The assessment of the studies’ methodological quality is shown in Supplementary Table 4 and the overall evaluation of the methodological quality is shown in Figure 4. The agreement of the two reviewers in assessing the methodological quality of included studies was 100%. The minority of the studies had good methodological quality in all domains, with the majority having low or unclear methodological quality. Using the GRADE approach [40], the certainty in evidence was rated to be very low due to the methodological limitations of the studies and imprecision of the estimates.

## Discussion

Pain is a common symptom of PC, and the frequency and severity of pain are associated with worse survival [7]. Although pharmacologic therapies are employed in pain management, narcotic medications may not provide adequate analgesia, requiring a frequent increase in the dose with a resultant increase in the risk of adverse drug effects [7]. Moreover, there are growing concerns regarding shortened survival with the increased use of narcotics [9] and CPN [10], which draws attention to an unmet clinical need for better treatment of PC pain.

Current paradigms in cancer pain management follow the four-step ‘analgesic ladder’ [41]. This approach, however, is mainly designed for the treatment of neuropathic pain patterns. For example, the fourth step (for patients with severe pain) includes interventions like CPN, whether via EUS or interventional radiology approaches, and such procedures are well accepted and advocated as part of the guidelines [42]. Other modalities of PC pain management include pancreatic-enzyme-replacement therapy, which is reserved mainly for patients with pancreatic exocrine insufficiency induced by the underlying malignancy [43]. However, such therapies may not address the post-prandial obstructive-type pain.

While PD stenting is a widely accepted practice for chronic-pancreatitis obstructive-type pain, it is often not considered for malignant strictures, although it has been suggested by the British Society of Gastroenterology guidelines for PC pain management [44]. In this systematic review, we studied a highly selected cohort of PC patients with what the authors deemed to be obstructive-type pain. Although the meta-analysis shows that PD stenting may be a safe and effective procedure in the majority (93%) of such carefully selected patients, and although the relief was maintained for a mean duration of 94 days, these results should be exploratory or hypothesis-generating, given the unclear or low methodological quality of the majority of these studies. The high success rate is likely a function of a substantial selection bias, which is desired in this context, as we found that patients whose pain pattern was explicitly suggestive of an obstructive cause appeared to benefit from this invasive intervention.

In PC, abdominal pain may be broadly divided into neuropathic pain and obstructive pain. The former has been described as a continuous, dull aching pain in the epigastrium or upper back, which is unrelated to meals. A similar neuropathic character of pain has been described in patients with chronic pancreatitis [45]. Obstructive pain in patients with PC is often described as intermittent epigastric pain radiating to the back, triggered by meals, and potentially due to ductal hypertension [46]. One study showed the importance of patient selection and compared the efficacy of PD stenting in PC patients with obstructive-type pain to its efficacy in patients with chronic unremitting pain; while 75% of patients with obstructive-type PC pain improved with PD stenting, none of those with chronic unremitting pain benefited when a similar intervention was performed [38]. This observation was also shown by Gao *et al.* [37], wherein stents placed for PC patients with PD dilation resulted in pain improvement with decreased visual analog scale scores in 74% of patients at 1 month; however, only 16% of PC patients who underwent PD stenting without ductal dilation at baseline had improvement in pain scores.

The increased ductal pressure may lead to ductal leakage and parenchymal infiltration by pancreatic juice [47], resulting in a compartment-syndrome-like picture within the pancreas and leading to ischemic pain. There is considerable evidence showing pain relief in patients with chronic pancreatitis and ductal obstruction who undergo PD stenting. In these patients, the European Society of Gastrointestinal Endoscopy recommends PD stenting as the first-line therapy for pain relief [48], with good efficacy in the immediate and long terms (pain relief in 88% and 67%, respectively, in a recent meta-analysis [49]). One study described the complete disappearance of pain in 60% and a significant reduction in analgesic medication in 20%–25% of PC patients who underwent PD stenting [3]. Another study showed that patients who underwent both biliary and PD stenting had better pain outcomes compared with those with strictures in the tail region [30, 37]. In the study by Wehrmann *et al.* [39], PD stenting was considered only in patients with pancreatic-head cancer. Interestingly, this predilection for patient selection and response, when the stricture is in the pancreatic head, is also observed in the case of chronic-pancreatitis ductal-obstruction pain [50]. However, stent placement in the body/tail of the pancreas is more technically challenging and may have lower technical success rates [50].

It should also be noted that acute pancreatitis can be a complication of PC, which can lead to severe acute pain, and may occur in 6%–14% of patients [51–53]. However, in this context, pancreatitis is usually mild [53]. Therefore, we excluded studies that implemented PD stenting to manage acute pancreatitis in PC.

Although our study suggests that PD stenting in selected patients with PC may be effective, this intervention is challenging and should only be pursued by an experienced endoscopist at a high-volume center, similar to what is required for pancreatic interventions in general. Before the procedure, the ductal anatomy needs to be delineated by cross-sectional imaging, or EUS, to localize the stricture accurately and to rule out ductal anomalies, such as pancreas divisum, seen in 7% of patients and which would warrant ductal access via the minor papilla [54–56]. PD-stricture dilation is performed when a larger-diameter PD stent needs to be deployed [57]. Ideally, a soft 10 Fr stent with side-holes, or multiple side-by-side smaller-caliber stents, should be placed into the PD. The use of a single stent ≤5 Fr is not advised due to short patency rates [46]. Self-expanding metallic stents have been used for this indication successfully to prolong the patency [35, 36, 38], but their application may be limited by the size of the duct, as well as the theoretical risk of obstructing PD side-branches. No episodes of pancreatic necrosis or acute pancreatitis were reported in these patients.

PD stenting is associated with unique complications, such as acute pancreatitis, bleeding, ductal rupture, and cholangitis [34, 58–61]. The risk of PEP is known to be lower in patients undergoing PD stent placement [62] and lower in PC patients in general [63]. Our systematic review shows a PEP incidence of 0.6% in the examined cohort. Other early complications include post-sphincterotomy bleeding in three patients (1.8%) and hemosuccus pancreaticus in one patient (0.6%). Late complications include stent-related ductal and parenchymal changes, stent occlusion, and migration [58]. Other serious risks of PD interventions in this setting is the risk of infection and pancreatic-abscess development [64, 65], although none was identified in our study. On the other hand, there is a theoretical possibility that relief of obstruction could improve survival from a decrease in inflammation related to ductal decompression and improved digestion.

Our study has several shortcomings, which limits the quality of this review and may lead to uncertainty of the conclusion. First, no RCTs were identified in the systematic review and the meta-analysis is unable to quantify the degree of pain improvement mainly due to the low utility of validated pain score assessments in the included studies, which are mostly case series and case reports with a small number of patients. Furthermore, the placebo effect may also be a possibility given the non-comparative nature of the majority of the included studies. Second, missing data precluded gathering complete information and not all patients were followed until their demise to establish the durability of the therapy. Third, substantial selection bias exists, although this should be viewed critically, and perhaps favorably, since such invasive therapy should only be offered to PC patients with PD obstruction who are not considered surgical candidates. However, there were no systematic or precise inclusion criteria explicitly stated in most of the studies. Fourth, other confounding factors such as the use of concomitant chemotherapy, which has been shown to improve pain relief and the quality of life in PC patients [66], were not described in detail to delineate a relationship, if one existed, during PD stenting. We attempted to compensate for some of these limitations by following a controlled *a priori* protocol and rigorous methodological quality appraisal.

Another main limitation is the inability of our meta-narrative evaluation to precisely estimate the AEs rate when the technique is applied. Although we reported all purported events as relayed in the original publications, uncertainty remains regarding the denominator used in three of the original reports [3, 37, 39]. In an attempt to compensate for this shortcoming, we included all events and related them to the subgroup of patients who fulfilled our inclusion criteria, so as not to underestimate the risk of the procedure.

Although this is the first systematic study that synthesizes all available evidence on the role of PD stenting in PC abdominal pain palliation, our results are inconclusive and instead suggest that a well-designed RCT is warranted, with numerous other questions remaining. This includes the most appropriate type and size of stents, the route of stent insertion (traditional transpapillary vs EUS-guided drainage), response according to tumor location, the appropriate interval of stent exchange, and the role of multiple stents. Patients with a PD stricture might also have underlying neuropathic pain, which will need to be addressed by other interventions.

In conclusion, for selected PC patients with obstructive-type pain symptoms and PD obstruction, PD stent placement may be an effective palliative approach, with an acceptable AEs profile. However, this systematic review and meta-analysis were based on the limited available literature of nonrandomized and retrospective data, with very low certainty in the evidence. While the analysis is exploratory, conclusive evidence should be derived from a larger-scale, well-executed, and adequately powered prospective randomized–controlled trial to corroborate these findings and help a subset of patients suffering from a highly morbid sequela of PC.

## Supplementary data

Supplementary data is available at *Gastroenterology Report* online. 

## Authors’ contributions

P.S., F.H., and L.J.P. collected the data. S.P., F.H., F.B., and M.H.M. analysed and interpreted the data. F.B. conceptualized and designed the study. S.P. and F.H. drafted the manuscript. L.J.P., M.H.M., B.K.A.D., V.C., M.D.T., and F.B. interpreted the data and critically revised the manuscript. All authors read and approved the final manuscript.

## Funding

No specific grant was received from any public, commercial, or not-for-profit sectors for this research activity. 

## Conflicts of interest

V.C.: Shareholder: Nevakar corporation, Consultant: Interpace Diagnostics. B.K.A.D.: Consultant: Metamodix, BFKW, DyaMx, Boston Scientific, USGI Medical, Hemostasis. Research support: Apollo Endosurgery, USGI, Spatz Medical, Boston Scientific, GI Dynamics, Cairn Diagnostics, Aspire Bariatrics, Medtronic. Speaker: Johnson and Johnson, Endogastric solutions, Olympus. The remaining authors declare that there is no conflict of interest in this study.

## Supplementary Material

goab001_Supplementary_Data

## References

[1] Siegel RL , Miller KD, Jemal A. Cancer statistics, 2020. CA A Cancer J Clin 2020;70:7–30. 10.3322/caac.21590 31912902

[2] Allen PJ , Kuk D, Castillo CF et al Multi-institutional validation study of the American Joint Commission on Cancer changes for T and N staging in patients with pancreatic adenocarcinoma. Ann Surg 2017;265:185–91. 27163957 10.1097/SLA.0000000000001763 PMC5611666

[3] Costamagna G , Alevras P, Palladino F et al Endoscopic pancreatic stenting in pancreatic cancer. Can J Gastroenterol Hepatol 1999;13:481–7. 10.1155/1999/123210 10464348

[4] Agarwal B , Krishna NB, Labundy JL el al EUS and/or EUS-guided FNA in patients with CT and/or magnetic resonance imaging findings of enlarged pancreatic head or dilated pancreatic duct with or without a dilated common bile duct. Gastrointest Endosc 2008;68:237–335. 18423464 10.1016/j.gie.2008.01.026

[5] Kanno A , Masamune A, Hanada K et al Multicenter study of early pancreatic cancer in Japan. Pancreatology 2018;18:61–7. 29170051 10.1016/j.pan.2017.11.007

[6] Vareedayah AA , Alkaade S, Taylor JR. Pancreatic adenocarcinoma. Mo Med 2018;115:230–5. 30228728 PMC6140147

[7] Koulouris AI , Banim P, Hart AR. Pain in patients with pancreatic cancer: Prevalence, mechanisms, management and future developments. Dig Dis Sci 2017;62:861–70. 28229252 10.1007/s10620-017-4488-z

[8] Moningi S , Walker AJ, Hsu CC et al Correlation of clinical stage and performance status with quality of life in patients seen in a pancreas multidisciplinary clinic. J Oncol Pract 2015;11:e216-21–e221. 25563703 10.1200/JOP.2014.000976 PMC4811042

[9] Novy DM , Nelson DV, Koyyalagunta D et al Pain, opioid therapy, and survival: a needed discussion. Pain 2020;161:496–501. 31693537 10.1097/j.pain.0000000000001736 PMC7017938

[10] Levy MJ , Gleeson FC, Topazian MD et al Combined celiac ganglia and plexus neurolysis shortens survival, without benefit, vs plexus neurolysis alone. Clin Gastroenterol and Hepatol 2019;17:728–38.e9. 10.1016/j.cgh.2018.08.040 30217513

[11] Kanno Y , Koshita S, Masu K et al Efficacy of EUS-guided celiac plexus neurolysis compared with medication alone for unresectable pancreatic cancer in the oxycodone/fentanyl era: a prospective randomized control study. Gastrointest Endosc 2020;92:120–30. 31953188 10.1016/j.gie.2020.01.011

[12] Lehman GA. Role of ERCP and other endoscopic modalities in chronic pancreatitis. Gastrointest Endosc 2002;56:S237–40. 12447274 10.1067/mge.2002.129008

[13] Rösch T , Daniel S, Scholz M, for the European Society of Gastrointestinal Endoscopy Research Group et al Endoscopic treatment of chronic pancreatitis: a multicenter study of 1000 patients with long-term follow-up. Endoscopy 2002;34:765–71. 12244496 10.1055/s-2002-34256

[14] Dite P , Ružicka M, Zboril V et al A prospective, randomized trial comparing endoscopic and surgical therapy for chronic pancreatitis. Endoscopy 2003;35:553–8. 12822088 10.1055/s-2003-40237

[15] Cahen DL , Gouma DJ, Laramée P et al Long-term outcomes of endoscopic vs surgical drainage of the pancreatic duct in patients with chronic pancreatitis. Gastroenterology 2011;141:1690–5. 21843494 10.1053/j.gastro.2011.07.049

[16] Moher D , Liberati A, Tetzlaff J et al Preferred reporting items for systematic reviews and meta-analyses: the PRISMA statement. Ann Intern Med 2009;151:264–9. 19622511 10.7326/0003-4819-151-4-200908180-00135

[17] Tenner S , Baillie J, DeWitt J, American College of Gastroenterology et al American College of Gastroenterology guideline: management of acute pancreatitis. Am J Gastroenterol 2013;108:1400–15. 23896955 10.1038/ajg.2013.218

[18] Treede R-D. The International Association for the Study of Pain definition of pain: as valid in 2018 as in 1979, but in need of regularly updated footnotes. Pain Rep 2018;3:e643. 29756089 10.1097/PR9.0000000000000643 PMC5902252

[19] Murad MH , Sultan S, Haffar S et al Methodological quality and synthesis of case series and case reports. Bmj Evid Based Med 2018;23:60–3. 10.1136/bmjebm-2017-110853 PMC6234235 29420178

[20] Jawoosh M , Haffar S, Deepak P et al Volvulus of the ileal pouch-anal anastomosis: a meta-narrative systematic review of frequency, diagnosis, and treatment outcomes. Gastroenterol Rep (Oxf) 2019;7:403–10. 31857902 10.1093/gastro/goz045 PMC6911998

[21] Haffar S , Kaur RJ, Garg SK et al Acute pancreatitis associated with intravenous administration of propofol: evaluation of causality in a systematic review of the literature. Gastroenterol Rep (Oxf) 2019;7:13–23. 30792862 10.1093/gastro/goy038 PMC6375349

[22] Bazerbachi F , Haffar S, Wang Z et al Range of normal liver stiffness and factors associated with increased stiffness measurements in apparently healthy individuals. Clin Gastroenterol Hepatol 2019;17:54–64.e1. 30196155 10.1016/j.cgh.2018.08.069

[23] Li DK , Haffar S, Horibe M et al Verrucous esophageal carcinoma is a unique indolent subtype of squamous cell carcinoma: a systematic review and individual patient regression analysis. J Gastroenterol 2020, doi:10.1007/s00535-020-01736-1. 33079233

[24] Bazerbachi F , Leise MD, Watt KD et al Systematic review of mixed cryoglobulinemia associated with hepatitis E virus infection: association or causation? Gastroenterol Rep (Oxf) 2017;5:178–84. 28852522 10.1093/gastro/gox021 PMC5554391

[25] Li DK , Rehan Khan M, Wang Z et al Normal liver stiffness and influencing factors in healthy children: an individual participant data meta-analysis. Liver Int 2020, doi:10.1111/liv.14658. 32901449

[26] Freeman MF , Tukey JW. Transformations related to the angular and the square root. Ann Math Stat 1950;21:607–11.

[27] Jackson D , Bowden J, Baker R. How does the DerSimonian and Laird procedure for random effects meta-analysis compare with its more efficient but harder to compute counterparts? J Stat Plan Inference 2010;140:961–70.

[28] Hunter JP , Saratzis A, Sutton AJ et al In meta-analyses of proportion studies, funnel plots were found to be an inaccurate method of assessing publication bias. J Clin Epidemiol 2014;67:897–903. 24794697 10.1016/j.jclinepi.2014.03.003

[29] Li Y , Dai J, Yang MR et al Effect of pancreatic stenting in relief of abdominal pain in advanced pancreatic cancer patients with pancreatic duct dilation. Shi Jie Hua Ren Xiao Hua Za Zhi 2016;24:2248–52.

[30] Catalano MF , Alcocer E, Raijman I et al Palliative endoscopic therapy in patients with pancreatic cancer and obstruction of the pancreatic duct using pancreatic stenting. Gastrointest Endosc 1998;47:AB133.

[31] Nair K , Mekaroonkamol P, Chawla S et al Pancreatic ductal stent placement for the palliation of pain in patients with pancreatic adenocarcinoma, with and without pancreatic ductal dilatation: 215. Am J Gastroenterol 2015;110:S94–5.

[32] ENDO 2017 Poster Presentations. Digestive Endoscopy 2017;29: 29–261, doi:10.1111/den.12775. 28120386

[33] Harrison MA , Hamilton JW. Palliation of pancreatic cancer pain by endoscopic stent placement. Gastrointest Endosc 1989;35:443–5. 2477301 10.1016/s0016-5107(89)72855-8

[34] Ashby K , Lo SK. The role of pancreatic stenting in obstructive ductal disorders other than pancreas divisum. Gastrointest Endosc 1995;42:306–11. 8536897 10.1016/s0016-5107(95)70127-3

[35] Akbar A , Baron TH. Covered self-expanding metal stent use in the pancreatic duct: a case series. Endoscopy 2012;44:869–73. 22752885 10.1055/s-0032-1309835

[36] Abramyan O , LaRusso NF, Tabibian JH. Pancreatobiliary ductal dilatation: unique pathobiological processes and endoscopic revelations. Gastroenterology 2019;156:876–8. 30612998 10.1053/j.gastro.2018.10.041

[37] Gao F , Ma S, Zhang N et al Clinical efficacy of endoscopic pancreatic drainage for pain relief with malignant pancreatic duct obstruction. Asian Pac J Cancer Prev 2014;15:6823–7. 25169532 10.7314/apjcp.2014.15.16.6823

[38] Tham TC , Lichtenstein DR, Vandervoort J et al Pancreatic duct stents for “obstructive type” pain in pancreatic malignancy. Am J Gastroenterol 2000;95:956–60. 10763944 10.1111/j.1572-0241.2000.01975.x

[39] Wehrmann T , Riphaus A, Frenz MB et al Endoscopic pancreatic duct stenting for relief of pancreatic cancer pain. Eur J Gastroenterol Hepatol 2005;17:1395–400. 16292095 10.1097/00042737-200512000-00020

[40] Murad MH. Clinical practice guidelines: a primer on development and dissemination. Mayo Clin Proc 2017;92:423–33. 28259229 10.1016/j.mayocp.2017.01.001

[41] Dobosz Ł , Stefaniak T, Dobrzycka M et al Invasive treatment of pain associated with pancreatic cancer on different levels of WHO analgesic ladder. BMC Surg 2016;16:20. 27090728 10.1186/s12893-016-0136-3 PMC4836189

[42] Wyse JM , Battat R, Sun S et al Practice guidelines for endoscopic ultrasound-guided celiac plexus neurolysis. Endosc Ultrasound 2017;6:369–75. 29251270 10.4103/eus.eus_97_17 PMC5752758

[43] Brennan GT , Saif MW. Pancreatic enzyme replacement therapy: a concise review. J Oncol Pract 2019;20:121–5. PMC6858980 31736680

[44] Pancreatic Section, British Society of Gastroenterology, Pancreatic Society of Great Britain and Ireland et al Guidelines for the management of patients with pancreatic cancer periampullary and ampullary carcinomas. Gut 2005;54:v1–16. Suppl 15888770 10.1136/gut.2004.057059 PMC1867803

[45] Anderson MA , Akshintala V, Albers KM et al Mechanism, assessment and management of pain in chronic pancreatitis: recommendations of a multidisciplinary study group. Pancreatology 2016;16:83–94. 26620965 10.1016/j.pan.2015.10.015 PMC4761301

[46] Costamagna G , Mutignani M. Pancreatic stenting for malignant ductal obstruction. Dig Liv Dis 2004;36:635–8. 10.1016/j.dld.2004.05.001 15460850

[47] Sharaiha RZ , Widmer J, Kahaleh M. Palliation of pancreatic ductal obstruction in pancreatic cancer. Gastrointest Endosc Clin N Am 2013;23:917–23. 24079797 10.1016/j.giec.2013.06.010

[48] Dumonceau JM , Delhaye M, Tringali A et al Endoscopic treatment of chronic pancreatitis: European Society of Gastrointestinal Endoscopy (ESGE) Guideline–Updated August 2018. Endoscopy 2019;51:179–93. 30654394 10.1055/a-0822-0832

[49] Jafri M , Sachdev A, Sadiq J et al Efficacy of endotherapy in the treatment of pain associated with chronic pancreatitis: a systematic review and meta-analysis. J Oncol Pract 2017;18:125–32. PMC5619873 28966569

[50] Tringali A , Bove V, Vadalà di Prampero SF et al Long-term follow-up after multiple plastic stenting for refractory pancreatic duct strictures in chronic pancreatitis. Endoscopy 2019;51:930–5. 31378858 10.1055/a-0959-6163

[51] Kimura Y , Kikuyama M, Kodama Y. Acute pancreatitis as a possible indicator of pancreatic cancer: the importance of mass detection. Intern Med 2015;54:2109–14. 26328633 10.2169/internalmedicine.54.4068

[52] Köhler H , Lankisch PG. Acute pancreatitis and hyperamylasaemia in pancreatic carcinoma. Pancreas 1986;2:117–9. 10.1097/00006676-198701000-00018 2437571

[53] Li S , Tian B. Acute pancreatitis in patients with pancreatic cancer: timing of surgery and survival duration. Medicine (Baltimore) 2017;96:e5908. 28099352 10.1097/MD.0000000000005908 PMC5279097

[54] Smanio T. Proposed nomenclature and classification of the human pancreatic ducts and duodenal papillae: study based on 200 post mortems. Int Surg 1969;52:125–41. 5793091

[55] Stimec B , Bulajić M, Korneti V et al Ductal morphometry of ventral pancreas in pancreas divisum: comparison between clinical and anatomical results. Ital J Gastroenterol 1996;28:76–80. 8781998

[56] Dumonceau J-M , Devière J, Le Moine O et al Endoscopic pancreatic drainage in chronic pancreatitis associated with ductal stones: long-term results. Gastrointest Endosc 1996;43:547–55. 8781931 10.1016/s0016-5107(96)70189-x

[57] Ahmad J , Martin J. Pancreatic duct strictures. Curr Treat Options Gastro 2000;3:371–84. 10.1007/s11938-000-0052-5 11096598

[58] Smits ME , Badiga SM, Rauws EA et al Long-term results of pancreatic stents in chronic pancreatitis. Gastrointest Endosc 1995;42:461–7. 8566639 10.1016/s0016-5107(95)70051-x

[59] Cremer M , Deviere J, Delhaye M et al Stenting in severe chronic pancreatitis: results of medium-term follow-up in seventy-six patients. Endoscopy 1991;23:171–6. 1860448 10.1055/s-2007-1010649

[60] Freeman ML , Overby C, Qi D. Pancreatic stent insertion: consequences of failure and results of a modified technique to maximize success. Gastrointest Endosc 2004;59:8–14. 14722540 10.1016/s0016-5107(03)02530-6

[61] Ponchon T , Bory RM, Hedelius F et al Endoscopic stenting for pain relief in chronic pancreatitis: results of a standardized protocol. Gastrointest Endosc 1995;42:452–6. 8566637 10.1016/s0016-5107(95)70049-8

[62] Tarnasky PR , Palesch YY, Cunningham JT et al Pancreatic stenting prevents pancreatitis after biliary sphincterotomy in patients with sphincter of Oddi dysfunction. Gastroenterology 1998;115:1518–24. 9834280 10.1016/s0016-5085(98)70031-9

[63] Matsubayashi H , Fukutomi A, Kanemoto H et al Risk of pancreatitis after endoscopic retrograde cholangiopancreatography and endoscopic biliary drainage. HPB (Oxford*)* 2009;11:222–8. 19590651 10.1111/j.1477-2574.2008.00020.x PMC2697892

[64] Kim MH , Kim GH, Kim SK et al A case of a pancreatic abscess complicating endoscopic sphincterotomy. Korean J Gastrointest Endosc 2009;39:55–8.

[65] Tseng A , Sales D, Simonowitz D et al Pancreas abscess: a fatal complication of endoscopic cholangiopancreatography (ERCP). Endoscopy 1977;9:250–3. 590219 10.1055/s-0028-1098529

[66] Kristensen A , Vagnildhaug O, Grønberg B et al Does chemotherapy improve health-related quality of life in advanced pancreatic cancer? A systematic review. Critical Rev Oncol Hematol 2016;99:286–98. 26819138 10.1016/j.critrevonc.2016.01.006

