# Supplementary material for: Endoscopic pancreatic duct stenting for pain palliation in selected pancreatic cancer patients: a systematic review and meta-analysis
Source: Gastroenterol Rep (Oxf). 2021 Feb 3;9(2):105–14. doi: 10.1093/gastro/goab001 (PMC8128017; doi:10.1093/gastro/goab001)
Supplement: goab001_Supplementary_Data [file goab001_supplementary_data.docx]

**Supplementary File 1**. Data sources, search strategies and extracted variables

A comprehensive search of several databases from each database’s inception to October 7, 2020, any language was conducted. The databases included Ovid MEDLINE Epub Ahead of Print, Ovid Medline In-Process & Other Non-Indexed Citations, Ovid MEDLINE, Ovid EMBASE, Ovid Cochrane Central Register of Controlled Trials, Ovid Cochrane Database of Systematic Reviews, and Scopus. The search strategy was designed and conducted by an experienced methodologist with input from the study’s principle investigator. We also searched the first 300 entries of Google Scholar using the terms 'pancreatic duct stents' to identify unpublished cases. Controlled vocabulary supplemented with keywords was used to search for pancreatic cancer and pancreatic duct stent. The actual strategy is available from the reprint author. A manual review of the reference lists of relevant publications was done for additional publications

**Actual search strategies**

**Ovid**

Database(s): EBM Reviews - Cochrane Central Register of Controlled Trials September 2020, EBM Reviews - Cochrane Database of Systematic Reviews 2005 to October 7, 2020, Embase 1974 to 2020 October 07, Ovid MEDLINE(R) and Epub Ahead of Print, In-Process & Other Non-Indexed Citations and Daily 1946 to October 02, 2020
Search Strategy:

| **#** | **Searches** | **Results** |
| --- | --- | --- |
| 1 | exp Pancreatic Neoplasms/ | 226183 |
| 2 | ((pancreas or pancreatic or "islet cell*") adj5 (cancer* or carcinoma* or metastas* or tumor* or tumour* or neoplas* or adenoma* or incidentaloma* or malignan*)).ti,ab,hw,kw. | 267894 |
| 3 | 1 or 2 | 286305 |
| 4 | exp Pain/ | 1782484 |
| 5 | pain*.ti,ab,hw,kw. | 2380990 |
| 6 | 4 or 5 | 2794125 |
| 7 | 3 and 6 | 20500 |
| 8 | exp Stents/ | 261524 |
| 9 | (((endoscop* or transmural*) adj3 drain*) or stent*).ti,ab,hw,kw. | 359109 |
| 10 | 8 or 9 | 361489 |
| 11 | 7 and 10 | 1295 |
| 12 | (exp animals/ or exp nonhuman/) not exp humans/ | 11189386 |
| 13 | ((alpaca or alpacas or amphibian or amphibians or animal or animals or antelope or armadillo or armadillos or avian or baboon or baboons or beagle or beagles or bee or bees or bird or birds or bison or bovine or buffalo or buffaloes or buffalos or "c elegans" or "Caenorhabditis elegans" or camel or camels or canine or canines or carp or cats or cattle or chick or chicken or chickens or chicks or chimp or chimpanze or chimpanzees or chimps or cow or cows or "D melanogaster" or "dairy calf" or "dairy calves" or deer or dog or dogs or donkey or donkeys or drosophila or "Drosophila melanogaster" or duck or duckling or ducklings or ducks or equid or equids or equine or equines or feline or felines or ferret or ferrets or finch or finches or fish or flatworm or flatworms or fox or foxes or frog or frogs or "fruit flies" or "fruit fly" or "G mellonella" or "Galleria mellonella" or geese or gerbil or gerbils or goat or goats or goose or gorilla or gorillas or hamster or hamsters or hare or hares or heifer or heifers or horse or horses or insect or insects or jellyfish or kangaroo or kangaroos or kitten or kittens or lagomorph or lagomorphs or lamb or lambs or llama or llamas or macaque or macaques or macaw or macaws or marmoset or marmosets or mice or minipig or minipigs or mink or minks or monkey or monkeys or mouse or mule or mules or nematode or nematodes or octopus or octopuses or orangutan or "orang-utan" or orangutans or "orang-utans" or oxen or parrot or parrots or pig or pigeon or pigeons or piglet or piglets or pigs or porcine or primate or primates or quail or rabbit or rabbits or rat or rats or reptile or reptiles or rodent or rodents or ruminant or ruminants or salmon or sheep or shrimp or slug or slugs or swine or tamarin or tamarins or toad or toads or trout or urchin or urchins or vole or voles or waxworm or waxworms or worm or worms or xenopus or "zebra fish" or zebrafish) not (human or humans or patient or patients)).ti,ab,hw,kw. | 9680485 |
| 14 | 11 not (12 or 13) | 1290 |
| 15 | limit 14 to (editorial or erratum or note or addresses or autobiography or bibliography or biography or blogs or comment or dictionary or directory or interactive tutorial or interview or lectures or legal cases or legislation or news or newspaper article or overall or patient education handout or periodical index or portraits or published erratum or video-audio media or webcasts) [Limit not valid in CCTR,CDSR,Embase,Ovid MEDLINE(R),Ovid MEDLINE(R) Daily Update,Ovid MEDLINE(R) In-Process,Ovid MEDLINE(R) Publisher; records were retained] | 48 |
| 16 | 14 not 15 | 1242 |
| 17 | remove duplicates from 16 | 1035 |

**Scopus**

1 TITLE-ABS-KEY((pancreas or pancreatic or "islet cell*") W/5 (cancer* or carcinoma* or metastas* or tumor* or tumour* or neoplas* or adenoma* or incidentaloma* or malignan*))

2 TITLE-ABS-KEY(pain*)

3 TITLE-ABS-KEY(((endoscop* or transmural*) W/3 drain*) OR stent*)

4 1 and 2 and 3

5 TITLE-ABS-KEY((alpaca OR alpacas OR amphibian OR amphibians OR animal OR animals OR antelope OR armadillo OR armadillos OR avian OR baboon OR baboons OR beagle OR beagles OR bee OR bees OR bird OR birds OR bison OR bovine OR buffalo OR buffaloes OR buffalos OR "c elegans" OR "Caenorhabditis elegans" OR camel OR camels OR canine OR canines OR carp OR cats OR cattle OR chick OR chicken OR chickens OR chicks OR chimp OR chimpanze OR chimpanzees OR chimps OR cow OR cows OR "D melanogaster" OR "dairy calf" OR "dairy calves" OR deer OR dog OR dogs OR donkey OR donkeys OR drosophila OR "Drosophila melanogaster" OR duck OR duckling OR ducklings OR ducks OR equid OR equids OR equine OR equines OR feline OR felines OR ferret OR ferrets OR finch OR finches OR fish OR flatworm OR flatworms OR fox OR foxes OR frog OR frogs OR "fruit flies" OR "fruit fly" OR "G mellonella" OR "Galleria mellonella" OR geese OR gerbil OR gerbils OR goat OR goats OR goose OR gorilla OR gorillas OR hamster OR hamsters OR hare OR hares OR heifer OR heifers OR horse OR horses OR insect OR insects OR jellyfish OR kangaroo OR kangaroos OR kitten OR kittens OR lagomorph OR lagomorphs OR lamb OR lambs OR llama OR llamas OR macaque OR macaques OR macaw OR macaws OR marmoset OR marmosets OR mice OR minipig OR minipigs OR mink OR minks OR monkey OR monkeys OR mouse OR mule OR mules OR nematode OR nematodes OR octopus OR octopuses OR orangutan OR "orang-utan" OR orangutans OR "orang-utans" OR oxen OR parrot OR parrots OR pig OR pigeon OR pigeons OR piglet OR piglets OR pigs OR porcine OR primate OR primates OR quail OR rabbit OR rabbits OR rat OR rats OR reptile OR reptiles OR rodent OR rodents OR ruminant OR ruminants OR salmon OR sheep OR shrimp OR slug OR slugs OR swine OR tamarin OR tamarins OR toad OR toads OR trout OR urchin OR urchins OR vole OR voles OR waxworm OR waxworms OR worm OR worms OR xenopus OR "zebra fish" OR zebrafish) AND NOT (human OR humans or patient or patients))

6 4 and not 5

7 DOCTYPE(ed) OR DOCTYPE(bk) OR DOCTYPE(er) OR DOCTYPE(no) OR DOCTYPE(sh)

8 6 and not 7

9 INDEX(embase) OR INDEX(medline) OR PMID(0* OR 1* OR 2* OR 3* OR 4* OR 5* OR 6* OR 7* OR 8* OR 9*)

10 8 and not 9

**Final result:** 44

**Supplementary Table 1.** Tool for assessment of the methodological quality of case reports and case series (adapted from Murad *et al*. [1])

| Domains | Leading explanatory questions |
| --- | --- |
| Selection | Does the patient(s) represent(s) the entire experience of the researchers, or is the selection modality unclear to the extent that other patients with similar presentation may have been missed? |
| Ascertainment | 1. Was the exposure sufficiently ascertained?  2. Was the outcome sufficiently ascertained? |
| Causality | Were other plausible causes that may account for the observation ruled out beyond a reasonable doubt? |
| Reporting | Is the case(s) relayed with adequate details to allow other investigators to replicate the research or to permit practitioners to make inferences related to their practice? |

1. Murad MH, Sultan S, Haffar S et al. Methodological quality and synthesis of case series and case reports. *BMJ Evid Based Med* 2018; 23: 60-63.

**Supplementary Table 2.** Clinical decision-making to patient selection for pancreatic duct stent placement

| First author/year | Patients with technical Success | MPD stricture | Upstream PD dilation | Pain characteristic |
| --- | --- | --- | --- | --- |
| Harrison/1989 [1] | 1 | Yes | Yes | EAP |
| Ashby/1995 [2] | 5 | Yes | Yes | AP NOS |
| Catalano/1998 [3] | 15 | Yes | NR | AP NOS |
| Costamagna/1999 [4] | 34 | Yes | Yes | Post-prandial EAP |
| Tham/2000 [5] | 6 | Yes | Yes | Post-prandial EAP |
| Wehrmann/2005 [6] | 19 | Yes | Yes | Post-prandial EAP |
| Akbar/2012 [7] | 2 | Yes | Yes | AP NOS |
| Gao/2014 [8] | 38 | Yes | Yes | UAP |
| Nair/2015 [9] | 2 | Yes | Yes^a^ | EAP |
| Li/2016 [10] | 42 | Yes | Yes | AP NOS |
| Ryabov/2017 [11] | 2 | Yes | Yes | AP NOS |
| Abramyan/2019 [12] | 1 | Yes | Yes | EAP |

MPD, Main pancreatic duct; PD, Pancreatic duct; EAP, Epigastric abdominal pain; UAP, Upper abdominal pain; AP NOS, Abdominal pain, not otherwise specified.
^a^ Only 1 out of the 2 patients had upstream ductal dilation.

**References:**

1. Harrison MA, Hamilton JW. Palliation of pancreatic cancer pain by endoscopic stent placement. *Gastrointest Endosc* 1989; 35: 443-445.
2. Ashby K, Lo SK. The role of pancreatic stenting in obstructive ductal disorders other than pancreas divisum. *Gastrointest Endosc* 1995; 42: 306-311.
3. Catalano MF, Alcocer E, Raijman I et al. Palliative endoscopic therapy in patients with pancreatic cancer and obstruction of the pancreatic duct using pancreatic stenting. *Gastrointest Endosc* 1998; 47: AB133.
4. Costamagna G, Alevras P, Palladino F et al. Endoscopic pancreatic stenting in pancreatic cancer. *Can J Gastroenterol Hepatol* 1999; 13: 481-487.
5. Tham TC, Lichtenstein DR, Vandervoort J et al. Pancreatic duct stents for “obstructive type” pain in pancreatic malignancy. *Am J Gastroenterol*  2000; 95: 956-960.
6. Wehrmann T, Riphaus A, Frenz MB, et al. Endoscopic pancreatic duct stenting for relief of pancreatic cancer pain. *Eur J Gastroenterol Hepatol* 2005; 17:1395-1400.
7. Akbar A, Baron T. Covered self-expanding metal stent use in the pancreatic duct: a case series. *Endoscopy* 2012; 44: 869-873.
8. Gao F, Ma S, Zhang N et al. Clinical efficacy of endoscopic pancreatic drainage for pain relief with malignant pancreatic duct obstruction. *Asian Pac J Cancer Prev* 2014; 15: 6823-6827.
9. Nair K, Mekaroonkamol P, Chawla S et al. Pancreatic ductal stent placement for the palliation of pain in patients with pancreatic adenocarcinoma, with and without pancreatic ductal dilatation: 215. *Am J Gastroenterol* 2015; 110: S94-S95.
10. Li Y, Dai J, Yang MR et al. Effect of pancreatic stenting in relief of abdominal pain in advanced pancreatic cancer patients with pancreatic duct dilation. *Shi Jie Hua Ren Xiao Hua Za Zhi* 2016; 24: 2248-2252.
11. Ryabov K, Rudakova M. Endoscopic treatment of pain syndrome in pancreatic cancer. *Dig Endosc* 2017; 29: 195.
12. Abramyan O, LaRusso NF, Tabibian JH. Pancreatobiliary ductal dilatation: Unique pathobiological processes and endoscopic revelations. *Gastroenterology* 2019; 156: 876-878.

**Supplementary Table 3.** Visual analog scale pain scores pre and post-intervention at 1 month

| First author/year | Pre-ERCP | Post-ERCP (1 month) | *P* value |
| --- | --- | --- | --- |
| Wehrmann/2005 [1] | 6.7+0.9 | 3.1+1.4 | < 0.001 |
| Gao/2014 [2] | 6.3+1.7 | 3+1 | < 0.05 |
| Li/2016 [3] | 6.8+1.7 | 3.7+0.7 | < 0.05 |

ERCP, Endoscopic retrograde cholangiopancreatography.

**References:**

1. Wehrmann T, Riphaus A, Frenz MB et al. Endoscopic pancreatic duct stenting for relief of pancreatic cancer pain. *Eur J Gastroenterol Hepatol* 2005; 17: 1395-1400.
2. Gao F, Ma S, Zhang N et al. Clinical efficacy of endoscopic pancreatic drainage for pain relief with malignant pancreatic duct obstruction. *Asian Pac J Cancer Prev* 2014; 15: 6823-6827.
3. Li Y, Dai J, Yang MR et al. Effect of pancreatic stenting in relief of abdominal pain in advanced pancreatic cancer patients with pancreatic duct dilation. *Shi Jie Hua Ren Xiao Hua Za Zhi* 2016; 24: 2248-2252.

**Supplementary Table 4.** Assessment of methodological quality of included studies

**Selection:** (Patient selection)

1. Prospective or retrospective study that included all patients within a specified time frame (e.g., 1990-2000): Good methodological quality (GMQ)
2. If a study only states several cases without mentioning a time frame: Unclear

methodological quality (UMQ)

1. If a study showed that several cases were missed due to a change in inclusion criteria: Unclear methodological quality (UMQ)
2. Case reports (1-4 cases): Low methodological quality (LMQ) [1]

**Ascertainment 1:** (Is the pain addressed suggestive of pancreatic-ductal hypertension?)

1. If a study clearly defines the abdominal pain characteristics as post-prandial AND intermittent WITHOUT other types of pain: GMQ
2. If a study does not explicitly mention the characteristics of the abdominal pain OR the quality is not GMQ or LMQ: UMQ
3. If a study defines the abdominal pain as dull, gnawing, continuous/persistent AND unrelated to meals: LMQ

**Ascertainment 2:**(Is the degree of pain change captured with clarity?)

1. If a study utilized pain assessment scales to describe the improvement in pain levels post-intervention: GMQ
2. If a study states that all symptoms have improved post-intervention: UMQ
3. If a study states non-rated/scaled improvement of pain levels post-intervention: LMQ

**Causality:**(Is the intervention the sole agent of the result, beyond a reasonable doubt?)

1. If the study has a comparative arm to pancreatic duct drainage and it explicitly stated that no other palliative interventions were applied, during the study period, in the pancreatic stenting arm of the study such as biliary stenting, opioid analgesia, or chemotherapy/radiation therapy, and hence, pain relief is most likely solely attributed to pancreatic duct drainage beyond a reasonable doubt: GMQ
2. If the study had a single arm for pancreatic duct stenting and/or did not specifically mention whether additional palliative interventions were used or not: UMQ
3. If other pain palliation interventions were used at the same time or immediately after endoscopic pancreatic duct drainage such as biliary stenting, opioid analgesics, surgery, or chemotherapy/radiation therapy: LMQ

**Reporting:**

1. If a study mentions A) the locations of the stricture, B) type of stent used, and C) other relevant procedure details: GMQ
2. If a study mentions some but not all of the above procedural components in #1: UMQ
3. If a study does not mention any of the above procedural components in #1: LMQ [1] Case reports were deemed those up to 4 patients based on Abu-Zidan et al.
   Abu-Zidan FM, Abbas AK, Hefny AF. Clinical "case series": a concept analysis. *Afr Health Sci* 2012; 12: 557-562.

| First Author/Year | | Selection | Ascertainment 1 | | Ascertainment 2 | | Causality | Reporting |
| --- | --- | --- | --- | --- | --- | --- | --- | --- |
| Harrison/1989 [1] | 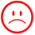 | | 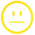 | 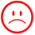 | | 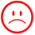 | | 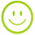 |
| Ashby/1995 [2] | 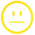 | | 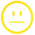 | 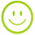 | | 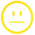 | | 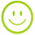 |
| Catalano/1998 [3] | 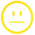 | | 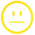 | 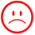 | | 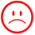 | | 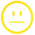 |
| Costamagna/1999 [4] | 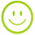 | | **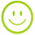** | 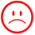 | | 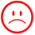 | | 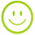 |
| Tham/2000 [5] | 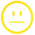 | | **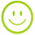** | 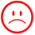 | | 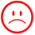 | | 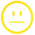 |
| Wehrmann/2005 [6] | 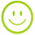 | | **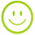** | 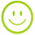 | | 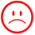 | | 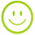 |
| Akbar/2012 [7] | 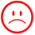 | | **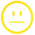** | 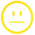 | | 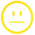 | | 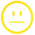 |
| Gao/2014 [8] | 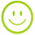 | | 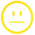 | 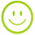 | | 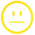 | | 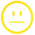 |
| Nair/2015 [9] | 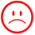 | | 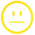 | 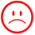 | | 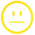 | | 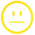 |
| Li/2016 [10] | 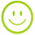 | | 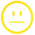 | 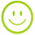 | | 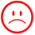 | | 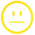 |
| Ryabov/2017 [11] | 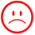 | | 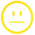 | 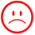 | | 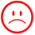 | | 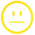 |
| Abramyan/2019 [12] | 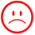 | | 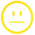 | 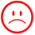 | | 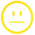 | | 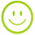 |
| Total: 12 Studies | **GMQ 4**  **UMQ 3**  **LMQ 5** | | **GMQ 3**  **UMQ 9**  **LMQ 0** | **GMQ 4**  **UMQ 1**  **LMQ 7** | | **GMQ 0**  **UMQ 5**  **LMQ 7** | | **GMQ 5**  **UMQ 7**  **LMQ 0** |


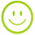
 Good methodological quality ;
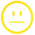
 Unclear methodological quality ;
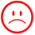
 Low methodological quality

GMQ, good methodological quality; UMQ, unclear methodological quality; LMQ, low methodological quality

**References:**

1. Harrison MA, Hamilton JW. Palliation of pancreatic cancer pain by endoscopic stent placement. *Gastrointest Endosc* 1989; 35: 443-445.
2. Ashby K, Lo SK. The role of pancreatic stenting in obstructive ductal disorders other than pancreas divisum. *Gastrointest Endosc* 1995; 42: 306-311.
3. Catalano MF, Alcocer E, Raijman I et al. Palliative endoscopic therapy in patients with pancreatic cancer and obstruction of the pancreatic duct using pancreatic stenting. *Gastrointest Endosc* 1998; 47: AB133.
4. Costamagna G, Alevras P, Palladino F et al. Endoscopic pancreatic stenting in pancreatic cancer. *Can J Gastroenterol Hepatol* 1999; 13: 481-487.
5. Tham TC, Lichtenstein DR, Vandervoort J et al. Pancreatic duct stents for “obstructive type” pain in pancreatic malignancy. *Am J Gastroenterol*  2000; 95: 956-960.
6. Wehrmann T, Riphaus A, Frenz MB, et al. Endoscopic pancreatic duct stenting for relief of pancreatic cancer pain. *Eur J Gastroenterol Hepatol* 2005; 17:1395-1400.
7. Akbar A, Baron T. Covered self-expanding metal stent use in the pancreatic duct: a case series. *Endoscopy* 2012; 44: 869-873.
8. Gao F, Ma S, Zhang N et al. Clinical efficacy of endoscopic pancreatic drainage for pain relief with malignant pancreatic duct obstruction. *Asian Pac J Cancer Prev* 2014; 15: 6823-6827.
9. Nair K, Mekaroonkamol P, Chawla S et al. Pancreatic ductal stent placement for the palliation of pain in patients with pancreatic adenocarcinoma, with and without pancreatic ductal dilatation: 215. *Am J Gastroenterol* 2015; 110: S94-S95.
10. Li Y, Dai J, Yang MR et al. Effect of pancreatic stenting in relief of abdominal pain in advanced pancreatic cancer patients with pancreatic duct dilation. *Shi Jie Hua Ren Xiao Hua Za Zhi* 2016; 24: 2248-2252.
11. Ryabov K, Rudakova M. Endoscopic treatment of pain syndrome in pancreatic cancer. *Dig Endosc* 2017; 29: 195.
12. Abramyan O, LaRusso NF, Tabibian JH. Pancreatobiliary ductal dilatation: Unique pathobiological processes and endoscopic revelations. *Gastroenterology* 2019; 156: 876-878.

**Supplementary Table 5.** Checklist of items in reporting a systematic review or meta-analysis (PRISMA statement)

| **Section/topic** | **Item** | **Checklist item** | **Reported on page** |
| --- | --- | --- | --- |
| **Title** | | | |
| Title | 1 | Identify the report as a systematic review, meta-analysis, or both | 1 |
| **Abstract** | | | |
| Structured summary | 2 | Provide a structured summary including, as applicable, background, objectives, data sources, study eligibility criteria, participants, interventions, study appraisal and synthesis methods, results, limitations, conclusions and implications of key findings, systematic review registration number | 3 |
| **Introduction** | | | |
| Rationale | 3 | Describe the rationale for the review in the context of what is already known | 4 |
| Objective | 4 | Provide an explicit statement of questions being addressed with reference to participants, interventions, comparisons, outcomes, and study design (PICOS) | 4 |
| **Methods** | | | |
| Protocol and registration | 5 | Indicate if a review protocol exists, if and where it can be accessed (such as web address), and, if available, provide registration information including registration number | 5 |
| Eligibility criteria | 6 | Specify study characteristics (such as PICOS, length of follow-up) and report characteristics (such as years considered, language, publication status) used as criteria for eligibility, giving rationale | 5 |
| Information sources | 7 | Describe all information sources (such as databases with dates of coverage, contact with study authors to identify additional studies) in the search and date last searched | 5 |
| Search | 8 | Present full electronic search strategy for at least one database, including any limits used, such that it could be repeated | 5 and supplementary file 1 |
| Study selection | 9 | State the process for selecting studies (that is, screening, eligibility, included in systematic review, and, if applicable, included in the meta-analysis) | 5 |
| Data collection process | 10 | Describe method of data extraction from reports (such as piloted forms, independently, in duplicate) and any processes for obtaining and confirming data from investigators | 6 and supplementary file 1 |
| Data items | 11 | List and define all variables for which data were sought (such as PICOS, funding sources) and any assumptions and simplifications made | N/A |
| Risk of bias in individual studies | 12 | Describe methods used for assessing risk of bias of individual studies (including specification of whether this was done at the study or outcome level), and how this information is to be used in any data synthesis | 7 and supplementary table 1 |
| Summary measures | 13 | State the principal summary measures (such as risk ratio, difference in means) | N/A |
| Synthesis of results | 14 | Describe the methods of handling data and combining results of studies, if done, including measures of consistency (such as I2 statistic) for each meta-analysis | 7 |
| Risk of bias across  studies | 15 | Specify any assessment of risk of bias that may affect the cumulative evidence (such as publication bias, selective reporting within studies) | 7 |
| Additional analyses | 16 | Describe methods of additional analyses (such as sensitivity or subgroup analyses, meta-regression), if done, indicating which were pre-specified | NA |
| **Results** | | | |
| Study selection | 17 | Give numbers of studies screened, assessed for eligibility, and included in the review, with reasons for exclusions at each stage, ideally with a flow diagram | 8 |
| Study characteristics | 18 | For each study, present characteristics for which data were extracted (such as study size, PICOS, follow-up period) and provide the citations | 8 and table 1 |
| Risk of bias within  studies | 19 | Present data on risk of bias of each study and, if available, any outcome-level assessment (see item 12). | 12, supplementary table 4, and Figure 4 |
| Results of individual  studies | 20 | For all outcomes considered (benefits or harms), present for each study (a) simple summary data for each intervention group and (b) effect estimates and confidence intervals, ideally with a forest plot | 8, 9, 10, 11 and  Table 1 and 2 |
| Synthesis of results | 21 | Present results of each meta-analysis done, including confidence intervals and measures of consistency | 11 |
| Risk of bias across  studies | 22 | Present results of any assessment of risk of bias across studies (see item 15) | 11, supplementary table 4, and figure 4 |
| Additional analysis | 23 | Give results of additional analyses, if done (such as sensitivity or subgroup analyses, meta-regression) (see  item 16) | N/A |
| **Discussion** | | | |
| Summary of evidence | 24 | Summarize the main findings including the strength of evidence for each main outcome; consider their relevance to key groups (such as health care providers, users, and policy makers) | 12, 13, 14, 15 |
| Limitations | 25 | Discuss limitations at study and outcome level (such as risk of bias), and at review level (such as incomplete retrieval of identified research, reporting bias) | 16 |
| Conclusions | 26 | Provide a general interpretation of the results in the context of other evidence, and implications for future research | 17 |
| **Funding** |  |  |  |
| Funding | 27 | Describe sources of funding for the systematic review and other support (such as supply of data) and role of funders for the systematic review | 18 |

N/A, Not applicable.

Modified from: Moher D, Liberati A, Tetzlaff J, Altman DG, PRISMA group. Preferred reporting items for systematic reviews and meta-analyses: the PRISMA statement. *Ann Intern Med* 2009; 151: 264-269.
